# Automated Pupillometry Identifies Absence of Intracranial Pressure Elevation in Intracerebral Hemorrhage Patients

**DOI:** 10.1007/s12028-020-01146-4

**Published:** 2020-12-24

**Authors:** Antje Giede-Jeppe, Maximilian I. Sprügel, Hagen B. Huttner, Matthias Borutta, Joji B. Kuramatsu, Philip Hoelter, Tobias Engelhorn, Stefan Schwab, Julia Koehn

**Affiliations:** 1 Department of Neurology, University of Erlangen-Nuremberg, Schwabachanlage 6, 91054 Erlangen, Germany; 2 Department of Neuroradiology, University of Erlangen-Nuremberg, Schwabachanlage 6, 91054 Erlangen, Germany

**Keywords:** Pupillary reactivity, Constriction velocity, Intracranial pressure, Intracerebral hemorrhage, Critical care

## Abstract

**Introduction:**

Although automated pupillometry is increasingly used in critical care settings, predictive value of automatically assessed pupillary parameters during different intracranial pressure (ICP) levels and possible clinical implications are unestablished.

**Methods:**

This retrospective cohort study at the neurocritical care unit of the University of Erlangen-Nuremberg (2016–2018) included 23 nontraumatic supratentorial (intracerebral hemorrhage) ICH patients without signs of abnormal pupillary function by manual assessment, i.e., absent light reflex. We assessed ICP levels by an external ventricular drain simultaneously with parameters of pupillary reactivity [i.e., maximum and minimum apertures, light reflex latency (Lat), constriction and redilation velocities (CV, DV), and percentage change of apertures (per-change)] using a portable pupillometer (NeurOptics®). Computed tomography (CT) scans were analyzed to determine lesion location, size, intraventricular hemorrhage, hydrocephalus, midline shift, and compression or absence of the basal cisterns. We performed receiver operating characteristics analysis to investigate associations of ICP levels with pupillary parameters and to determine best cutoff values for prediction of ICP elevation. After dichotomization of assessments according to ICP values (normal: < 20 mmHg, elevated: ≥ 20 mmHg), prognostic performance of the determined cutoff parameters of pupillary function versus of CT-imaging findings was analyzed by calculating sensitivity, specificity, positive and negative predictive values (logistic regression, corresponding ORs with 95% CIs).

**Results:**

In 23 patients (11 women, median age 59.0 (51.0–69.0) years), 1,934 assessments were available for analysis. A total of 74 ICP elevations ≥ 20 mmHg occurred in seven patients. Best discriminative thresholds for ICP elevation were: CV < 0.8 mm/s (AUC 0.740), per-change < 10% (AUC 0.743), DV < 0.2 mm/s (AUC 0.703), and Lat > 0.3 s (AUC 0.616). Positive predictive value of all four parameters to indicate ICP elevation ranged between 7.2 and 8.3% only and was similarly low for CT abnormalities (9.1%). We found high negative predictive values of pupillary parameters [CV: 99.2% (95% CI 98.3–99.6), per-change: 98.7% (95% CI 97.8–99.2), DV: 98.0% (95% CI 97.0–98.7), Lat: 97.0% (95% CI 96.0–97.7)], and CT abnormalities [99.7% (95% CI 99.2–99.9)], providing evidence that both techniques adequately identified ICH patients without ICP elevation.

**Conclusions:**

Our data suggest an association between noninvasively detected changes in pupillary reactivity and ICP levels in sedated ICH patients. Although automated pupillometry and neuroimaging seem not sufficient to noninvasively indicate ICP elevation, both techniques, however, adequately identified ICH patients without ICP elevation. This finding may facilitate routine management by saving invasive ICP monitoring or repeated CT controls in patients with specific automated pupillometry readings.

## Introduction

Although spontaneous intracerebral hemorrhage (ICH) represents a significant cause of morbidity and mortality throughout the world, standardized treatment protocols do not exist and management algorithms are often left to physicians' judgment [1, 2]. Due to high rates of neurological deterioration related to hematoma expansion, guidelines recommend frequent patient monitoring [1, 2]. In sedated patients on intensive care units (ICU) however, neurological assessments may have restricted informative value and therefore patients undergo repetitive neuroimaging. Furthermore, data on intracranial pressure (ICP) monitoring and treatment are limited [1, 2], and there are no randomized controlled trials clarifying possible benefits of invasive ICP monitoring in ICH patients [1, 2]. Thus, external ventricular drain (EVD) placement is mainly applied in patients with signs of hydrocephalus, while patients with mass lesions and absent hydrocephalus in clinical routine often do not undergo EVD placement, or ICP monitoring respectively, though being potentially at risk for ICP elevation [3].

For a time- and cost-effective patient care, reliable, noninvasive monitoring techniques would be desirable in order to distinguish between patients in need of repetitive imaging and invasive monitoring and those who might not require such procedures. Evaluation of pupillary function as an integral element of neurological assessment represents an easy bedside technique [3, 4]. While manual assessment of pupillary function may be subject to inaccuracy with a certain inter-rater variability [5] showing significant changes only in imminent herniation [5], automated pupillometry devices are now widely available providing an objective measure of the pupillary light reflex [4, 6]. Abnormalities of the pupillary response to light represent autonomic dysfunction at the level of central processing of the light reflex arc and have been linked to clinical deterioration and outcome [4, 7].

So far, only few studies assessed automated pupillometry for identification of patients with increased ICP [5, 8–10]; however, these studies included heterogeneous patient populations and only reported on correlations between decreasing pupillary parameters and increasing ICP levels. Hence, there is uncertainty on whether or not this technique is of any additional clinical value as there are no studies assessing prognostic performance of automated pupillometry in comparison with standard procedures. In order to establish the latter, we here determined the prognostic performance, i.e., sensitivity, specificity, positive and negative predictive values, of sympathetic and parasympathetic parameters of pupillary function compared to repetitive imaging and invasive ICP levels in ICH patients.

## Methods

### Patient Selection

All patients with nontraumatic supratentorial ICH admitted to the neurocritical care unit of the University Hospital Erlangen, Germany, between April 2016 and August 2018 were screened for eligibility to participate in the present retrospective study. Inclusion criteria consisted of (i) requirement of neurocritical care treatment on a certified ICU providing sedation and mechanical ventilation, (ii) necessity for EVD placement due to obstructive hydrocephalus, (iii) absent signs of abnormal pupillary function according to manual assessment, i.e., absent light reflex, and absence of trauma or structural eye abnormalities. For analyses, we only included measurements when ICP and pupillary parameters were assessed simultaneously with exact timestamps of documentation. Algorithms for treatment of ICP elevation were left to the judgment of the treating physicians. Yet, within the study cohort, only deep sedation was used to treat ICP elevation, while none of the participants received other therapies such as mannitol, hypertonic saline, or surgical interventions, i.e., hematoma evacuation or decompressive craniectomy. The institutional review board approved innocuousness of the study protocol.

### Data Assessment, CT Analysis, and Quantitative Pupillometry

We retrieved data on demographic parameters (age, sex), prior medical history, as well as clinical status on admission (Glasgow Coma Scale score [GCS], National Institute of Health Stroke Scale score [NIHSS]) from the institutional electronic databases. Diagnosis of ICH was made upon cranial computed tomography (CT) imaging (SOMATOM Definition AS +; Siemens Healthineers, Forchheim, Germany). CT scans conducted during patients' ICU stay were retrospectively analyzed by two specialized neuroradiologists in order to minimize a potential reporting bias and interobserver variability. These independent investigators were blinded to clinical parameters as well as to radiologic reports of the institutional electronic database. For data analysis, we used CT scans that were performed immediately upon hospital admission. Furthermore, we included neuroimaging data that were collected on the same day as assessments of ICP and pupillometry, i.e., with a maximum time difference of 24 h to the other assessments. Investigators scored ICH location and classified ICH as lobar or deep (arising in the basal ganglia or the thalamus) [11]. Hematoma volume was estimated using ABC methods (AxBxC/2) [12], and presence of intraventricular hemorrhage (IVH) was documented. Due to the fact that current management guidelines for ICH patients do not provide distinct recommendations about the indication for monitoring and treatment of ICP [1], we used CT features that have been associated with increased ICP in other head injuries (e.g., traumatic brain injury) [1]. Definition of CT abnormalities indicative of increased ICP consisted of (i) compression or absence of the basal cisterns [13], (ii) hydrocephalus (enlargement of the lateral ventricles measured as bicaudate index above the 95^th^ percentile for age) [14], and/or (iii) midline shift (displacement of the septum pellucidum, the pineal gland, or the aqueduct relative to the midline) > 5 mm [13]. According to the guidelines for the management of spontaneous ICH of 2015, ICP elevation was defined as ICP ≥ 20 mmHg [1], and ICP elevations of ≥ 3 min duration were documented for analyses. For standardization of ICP measurements, the EVD was clamped and calibrated for an ICP of zero in a supine position prior to each assessment. During calibration, the pressure transducer was placed in line with the Foramen of Monro, which refers to the level of the external auditory meatus of the ear and at the mid sagittal line (between the eyebrows) in the lateral position [15]. In order to minimize the potential bias that the same abnormality of ICP elevation affects repeated measurements, we only included several ICP elevations ≥ 20 mmHg in one patient when there was a delay of at least one hour between assessments.

Quantitative pupillometry was performed using the NeurOptics® pupillometer (NeurOptics, Irvine, CA, USA). The pupilometer uses an infrared camera that integrates a calibrated light stimulus of standardized intensity (1000 lx) and duration (3.2 s) [16]. The system automatically analyzes the following static and dynamic parameters over a 3-s time period: static: pupil size, i.e., maximum and minimum apertures (mm), dynamic: light reflex latency (s), constriction and redilation velocities (mm/s), and percentage change of apertures (%) [5]. The definitions of these parameters have been published previously [5]. Pupillary reactivity was monitored by the treating physicians or bedside nurses up to every 30 min for the duration of the ICU stay, and parameters of the eye ipsilateral to the lesion were documented for analyses.

### Statistical Analysis

A commercially available statistical program (IBM, SPSS Statistics 22) was used for data analysis. Significance was set at *p* < 0.05. We used the Kolmogorov–Smirnov test to test for normal distribution of data. Data are expressed as mean ± SD in the case of normal distribution or as median (and interquartile range) for variables with skewed distribution. For analysis of baseline characteristics and pupillary parameters, patients were categorized according to ICP values (< 20 mmHg, ≥ 20 mmHg). Descriptive statistics were computed for baseline characteristics and pupillary parameters using *t* tests for unpaired samples in the case of normally distributed variables and the nonparametric Mann–Whitney *U* test for unpaired samples in case of not normally distributed data. We compared frequency distributions of categorical variables (presented as counts [percentage]) by Pearson *χ*^2^ and Fisher’s exact tests. Receiver operating characteristics (ROC) analysis was performed to investigate associations of ICP levels with pupillary parameters and to determine the best cutoff values for prediction of ICP elevation. Subsequently, assessments were dichotomized according to ICP values (< 20 mmHg, ≥ 20 mmHg), and prognostic performance of determined pupillary parameters and CT-findings was analyzed by calculating sensitivity, specificity, positive and negative predictive values. Corresponding ORs with 95% CIs were calculated using logistic regression.

## Results

**Fig. 1:**
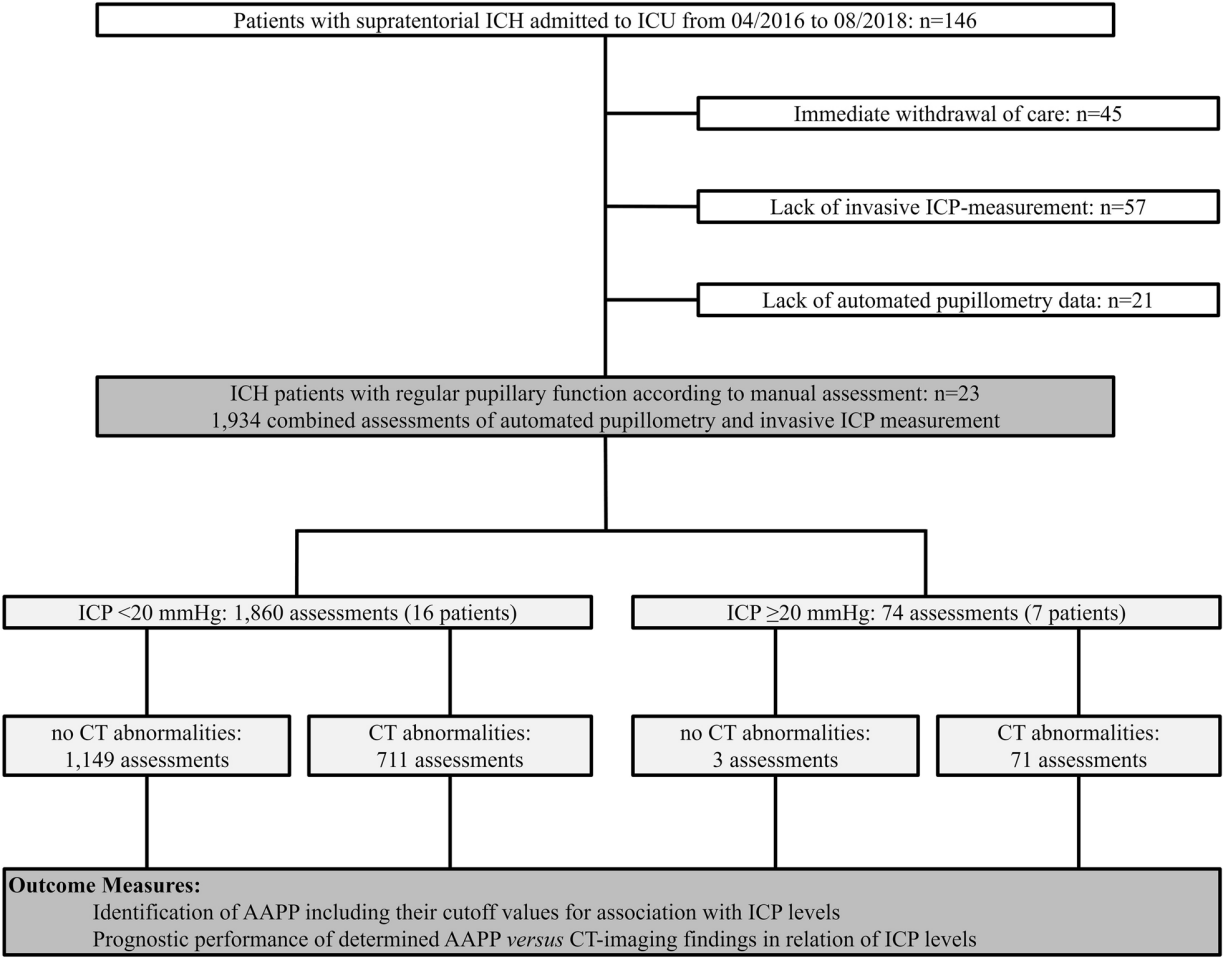
Flowchart of study participants. Overall, 146 patients with nontraumatic supratentorial ICH admitted to the ICU between April 2016 and August 2018 were screened for eligibility. After exclusion of 45 patients because of early care limitation, 57 patients because of lack of invasive ICP measurement, and 21 because of lack of automated pupillometry data, 23 ICH patients and 1,934 combined assessments of automated pupillometry and invasive ICP measurement were available for data analysis. AAPP, automatically assessed pupillary parameters; CT, computed tomography; ICH, intracerebral hemorrhage; ICP, intracranial pressure; ICU, intensive care unit

Over a 2.5-year period, a total of 23 nontraumatic supratentorial ICH patients (11 women, 12 men, median age 59.0 (51.0–69.0) years) without signs of abnormal, manually assessed pupillary function were enrolled (Fig. 1). There were 1,934 pupillary readings with simultaneously assessed ICP values available [median number of assessments per patient: 63 (38–107), minimum number of assessments: 5, maximum number of assessments: 331]. Clinical baseline characteristics of patients with and without elevated ICP are presented in Table 1. In 7/23 (30.4%) patients, a total of 74 ICP elevations were detected. Patients with ICP elevation were significantly younger (ICP elevation: 47.0 (30.0–63.0) years vs. 63.5 (55.5–73.0) years, *p* = 0.039), while there were no differences in other baseline clinical parameters (Table 1). In particular, parameters of clinical status on admission, i.e., GCS and NIHSS scores, did not differ between patients with and without ICP elevation (GCS: ICP < 20 mmHg: 3 (3–13), ICP ≥ 20 mmHg: 3 (3–8), *p* = 0.556; NIHSS: ICP < 20 mmHg: 29 (11–38), ICP ≥ 20 mmHg: 38 (13–38); *p* = 0.794).

**Table 1:** Baseline and clinical characteristics, of 23 patients with nontraumatic supratentorial intracerebral hemorrhage (ICH), according to intracranial pressure (ICP) values—either < 20 mm hg, or ≥ 20 mm hg

Intracranial hemorrhage (ICH) patients	ICP < 20 mmHg 16 patients	ICP ≥ 20 mmHg 7 patients	*p* value
Age, years, median (IQR)	63.5 (55.5–73.0)	47.0 (30.0–63.0)	0.039*
Gender	7 female, 9 male	4 female, 3 male	0.587
Prior comorbidities, N (%)			
Hypertension	9 (56.3%)	6 (85.7%)	0.145
Diabetes mellitus	1 (6.3%)	0 (0.0%)	0.333
Dyslipidemia	3 (18.8%)	0 (0.0%)	0.083
Prior myocardial infarction	0 (0.0%)	0 (0.0%)	
Congestive heart failure	3 (18.8%)	1 (14.3%)	0.803
Abnormal kidney function	0 (0.0%)	0 (0.0%)	
Prior ischemic stroke or TIA	2 (12.5%)	0 (0.0%)	0.164
Prior hemorrhagic stroke or major bleeding	0 (0.0%)	0 (0.0%)	
Premorbid mRS, median (IQR)	0 (0–1)	0 (0–0)	0.609
Admission status, mean (SD)			
Glasgow Coma Scale Score [GCS]	3 (3–13)	3 (3–8)	0.556
National Institute of Health Stroke Scale Score [NIHSS]	29 (11–38)	38 (13–38)	0.794
ICH characteristics, N (%)			
Deep	8 (50.0%)	7 (100.0%)	0.052
Lobar	8 (50.0%)	0 (0.0%)	0.052
Intraventricular hemorrhage	14 (87.5%)	7 (100.0%)	1.000
ICH score, median (IQR)	3 (1–4)	3 (2–3)	0.565
Intracranial hemorrhage (ICH) volume [ml], median (IQR)	12.0 (5.5–63.5)	15.4 (6.0–37.6)	0.274
Potentially confounding medication, N (%)			
Catecholamines	15 (93.8%)	6 (85.7%)	0.526
Benzodiazepines	15 (93.8%)	7 (100%)	1.000
Opioids	16 (100%)	7 (100%)	1.000
Other narcotics/anesthetics	15 (93.8%)	7 (100%)	1.000

*Indicates significant differences between patients with and without ICP elevation (ICP ≥ 20 mmHg); mRS score ranges from 0, no symptoms, to 6, death; GCS ranges from 3, comatose, to 15, alert; NIHSS ranges from 0, no deficit, to 40, severe neurological deficit (40 is the maximum because in comatose patients ataxia is not scored); ICH score ranges from 0 to 6, with higher scores indicating a higher probability of fatal outcome after ICH. GCS, Glasgow Coma Scale; ICH, intracerebral hemorrhage; ICP, intracranial pressure; mRS, modiied Rankin Scale; NIHSS, National Institutes of Health Stroke Scale; SD, standard deviation; TIA, transient ischemic attack

### Associations between Automatically Assessed Pupillary Parameters and ICP Levels

**Fig. 2:**
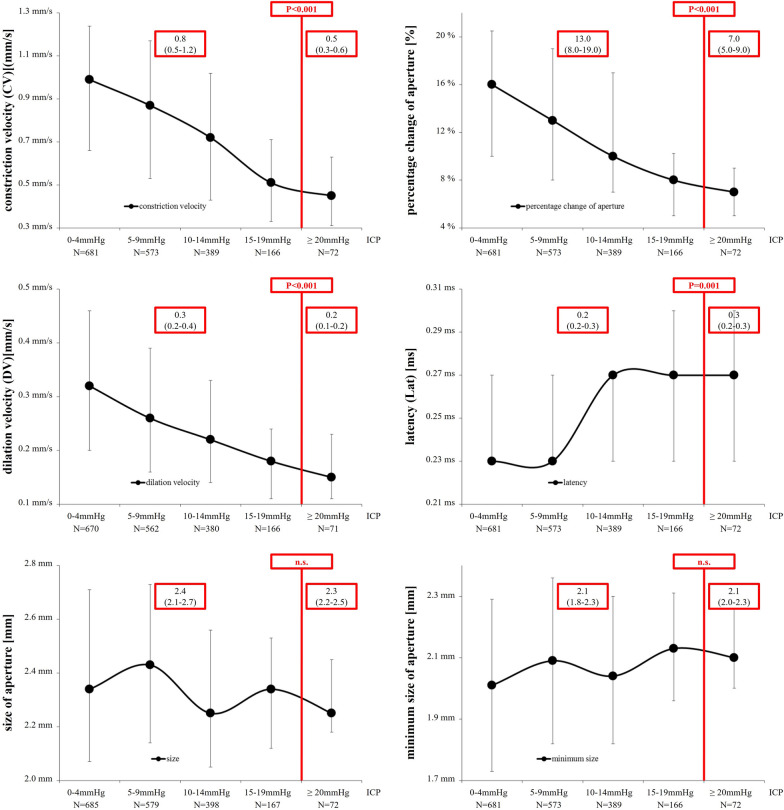
Automated pupillometry readings in relation to ICP levels. Constriction velocity (upper left graph), percentage change of aperture (upper right graph), dilation velocity (middle left graph), light reflex latency (middle right graph), size of aperture (lower left graph), and minimum size of aperture (lower right graph) according to intracranial pressure (ICP) values in 23 patients with nontraumatic supratentorial intracerebral hemorrhage (ICH; presented as median and interquartile range)

To investigate possible associations between automatically provided pupillary parameters and ICP levels, we compared dynamic and static pupillary parameters among patients with and without ICP elevation. All automatically provided dynamic pupillary parameters were significantly different between patients with and without ICP elevation (ICP elevation vs. no ICP elevation: constriction velocity: 0.5 mm/s (0.3–0.6) vs. 0.8 mm/s (0.5–1.2), *p* < 0.001; percentage change of aperture: 7.0% (5.0–9.0) vs. 13.0% (8.0–19.0), *p* < 0.001; dilation velocity: 0.2 mm/s (0.1–0.2) vs. 0.3 mm/s (0.2–0.4), *p* < 0.001; latency 0.3 s (0.2–0.3) vs. 0.2 s (0.2–0.3), *p* = 0.001; Fig. 2).

In the next step, we calculated cutoff values best discriminative for ICP elevation. While all four parameters showed significant associations between pupillary reactivity and ICP elevation, parasympathetic parameters showed higher AUC values for association with ICP elevation than sympathetic parameters (AUC [95% CI]): constriction velocity 0.740 (0.695–0.786), *p* < 0.001; percentage change of aperture 0.743 (0.697–0.788), *p* < 0.001; dilation velocity 0.703 (0.654–0.752); *p* < 0.001; latency 0.616 (0.544–0.689), *p* = 0.001). Best discriminative thresholds were: constriction velocity < 0.8 mm/s; percentage change of aperture < 10%; latency > 0.3 s; dilation velocity < 0.2 mm/s; Table 2.

**Table 2:** Receiver operating characteristics (ROC) analysis of pupillary parameters associated with intracranial pressure (ICP) level and prognostic value (i.e., sensitivity, specificity positive predictive value, negative predictive value, and false-negative rate) of pupillary parameters and CT findings for intracranial pressure (ICP) elevation ≥ 20 mmHg in 23 patients with nontraumatic supratentorial intracerebral hemorrhage (ICH)

	ROC analysis AUC (95% CI)	ROC analysis *p* value	ROC analysis calculated cutoff value	ICP < 20 mmHg (*n* = 1860)	ICP ≥ 20 mmHg (*n* = 74)	Sensitivity% (95% CI)	Specificity% (95% CI)	Positive predictive value% (95% CI)	Negative predictive value % (95% CI)	False-negative rate % (95% CI)
				Prognostic value of pupillary parameters (constriction velocity < 0.8 mm/s, percentage change of aperture < 10%, dilation velocity < 0.2 mm/s, light reflex latency > 0.3 s)						
CV	0.740 (0.695–0.786)	< 0.001	0.8 mm/s	837/1809 (46.3%)	65/73 (89.0%)	89.0 (79.0–94.8)	53.7 (51.4–56.0)	7.2 (5.6–9.1)	99.2 (98.3–99.6)	0.8 (0.4–1.7)
%	0.743 (0.697–0.788)	< 0.001	10.0%	625/1809 (34.5%)	57/73 (78.1%)	78.1 (66.6–86.6)	65.5 (63.2–67.6)	8.3 (6.4–10.8)	98.7 (97.8–99.2)	1.3 (0.8–2.2)
DV	0.703 (0.654–0.752)	< 0.001	0.2 mm/s	599/1778 (33.7%)	48/72 (66.7%)	66.7 (54.5–77.1)	66.3 (64.1–68.5)	7.4 (5.6–9.8)	98.0 (97.0–98.7)	2.0 (1.3–3.0)
Lat	0.616 (0.544–0.689)	0.001	0.3 s	307/1809 (17.0%)	26/73 (35.6%)	35.6 (25.0–47.8)	83.0 (81.2–84.7)	7.8 (5.3–11.4)	97.0 (96.0–97.7)	3.0 (2.3–4.0)
				Prognostic value of CT abnormalities (i.e., midline shift ≥ 5 mm, asymmetrical or absent basal cisterns)						
CT				711/1860 (38.2%)	71/74 (95.9%)	95.9 (87.8–98.9)	61.8 (59.5–64.0)	9.1 (7.2–11.4)	99.7 (99.2–99.9)	0.3 (0.1–0.8)

(ROC, Receiver operating characteristics analysis; AUC, area under the curve; CI, confidence interval; ICP, intracranial pressure; CV, constriction velocity, amount of constriction divided by the duration of the constriction [5]; %, percentage change of aperture, constriction percentage, i.e., maximum size minus minimum size divided by maximum size [5]; DV, dilation velocity, amount of pupil size recovery divided by the duration of the recovery [5]; Lat, light reflex latency, time difference between initiation of retinal light stimulation and onset of pupillary constriction [5]; CT, computed tomography). Due to the fact that not all pupillary parameters were available for the 1860 assessments, total numbers of CV, %, DV, and Lat are not identical

### Prognostic Value of Pupillary Parameters and CT-Imaging Findings for Identification of Patients *With ICP Elevation*

To predict ICP elevation, (i) constriction velocity < 0.8 mm/s provided a sensitivity of 89.0% and a specificity of 53.7% (OR [95% CI] 9.44 (4.50–19.78), *p* < 0.001), (ii) percentage change of aperture < 10% provided a sensitivity of 78.1% and a specificity of 65.5% (OR [95% CI] 6.75 (3.84–11.85), *p* < 0.001), (iii) dilation velocity < 0.2 mm/s provided a sensitivity of 66.7% and a specificity of 66.3% (OR [95% CI] 3.94 (2.39–6.49), *p* < 0.001), and (iv) latency > 0.3 s provided a sensitivity of 35.6% and a specificity of 83.0% (OR [95% CI] 2.71 (1.65–4.44), *p* < 0.001). As positive predictive values of all four parameters ranged between 7.2 and 8.3% only (Table 2), analysis of the prognostic value of CT abnormalities for ICP elevation demonstrated a sensitivity of 95.9% and a specificity of 61.8% (OR [95% CI] 38.25 (12.00–121.88), *p* < 0.001) with a similarly low positive predictive value of 9.1% (95% CI 7.2–11.4); Table 2), providing evidence that both techniques fail to indicate ICP elevation sufficiently.

### Prognostic Value of Pupillary Parameters and CT-Imaging Findings for Identification of Patients *Without ICP Elevation*

**Fig. 3:**
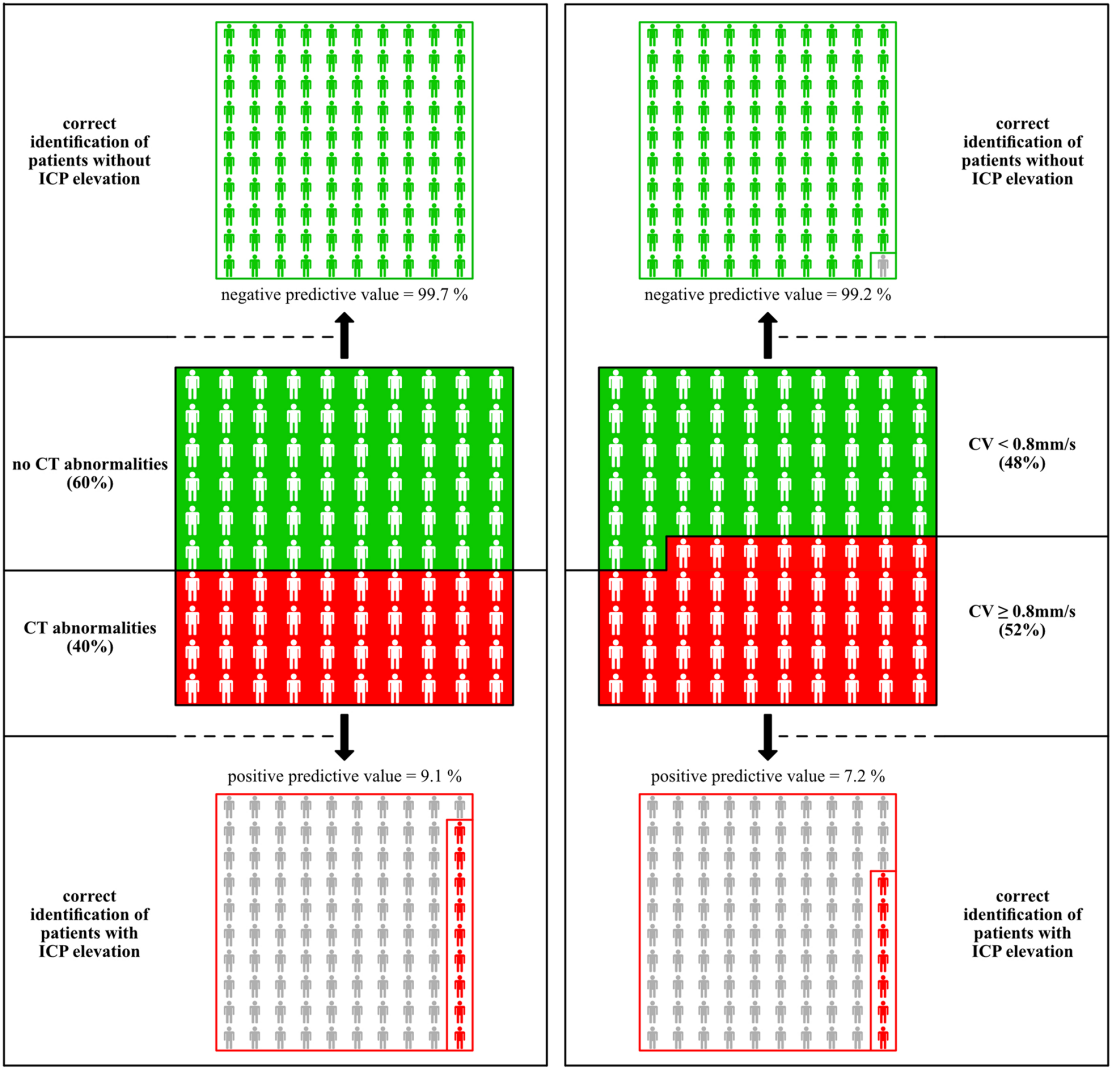
Prognostic performance of neuroimaging and automated pupillometry for identification of patients with normal *versus* elevated ICP levels. Hypothetical clinical scenario of 100 patients with supratentorial ICH monitored by CT imaging and automated pupillometry. Middle left and right graphs illustrate the percentage of ICH patients with (red background) and without (green background) CT abnormalities (left graph), respectively, with (red background) and without (green background) CV abnormalities (right graph). Prognostic relevance of abnormal neuroimaging and pupillometry findings for identification of ICP levels > 20 mmHg is demonstrated in the lower left and right graph. Positive predictive values of both monitoring techniques ranged less than 10% only, illustrated as red figures within the lower graphs. Gray figures visualize the high percentage of patients with CT abnormalities (left), respectively, CV abnormalities (right) despite ICP values below 20 mmHg. Prognostic relevance of both techniques for identification of ICP levels < 20 mmHg in case of absent abnormal neuroimaging and pupillometry findings is demonstrated in the upper left and right graph. Negative predictive values of CT findings (left, 99.7%) and CV (right, 99.2%) are illustrated as green figures, i.e., patients reliably identified as not at risk of ICP elevation. CT, computed tomography; CV, constriction velocity; ICP, intracranial pressure

Negative predictive values of pupillary parameters were: constriction velocity < 0.8 mm/s: 99.2% (95% CI 98.3–99.6); percentage change of aperture < 10%: 98.7% (95% CI 97.8–99.2); dilation velocity < 0.2 mm/s: 98.0% (95% CI 97.0–98.7); latency > 0.3 s: 97.0% (95% CI 96.0–97.7). Negative predictive value of CT imaging abnormalities was 99.7% (95% CI 99.2–99.9; Table 2). These high negative predictive values verified that both techniques adequately identified ICH patients without ICP elevation (Fig. 3), translating into a hypothetical clinical scenario of 100 ICH patients of whom 52 patients would be identified as having no ICP elevation (with constriction velocities above 0.8 mm/s). The high negative predictive value suggests that only one patient may be missed of being at risk of ICP elevation (Fig. 3).

## Discussion

We here demonstrate an additional clinical value of automated pupillometry over manual assessment of pupillary reactivity in identifying patients with normal *versus* elevated ICP levels. In essence, (i) all dynamic pupillary parameters showed a significant association with ICP and a decrease of pupillary modulation with increasing ICP levels. While (ii) automated pupillometry was not able to reliably determine patients with increased ICP, it (iii) robustly identified patients without ICP elevation, and (iv) specifically parasympathetic parameters appear to most robustly indicate absent increased ICP. Some aspects emerge from the data.

Although available for decades, in recent years automated pupillometry has gained increasing attention from critical care physicians given next-generation devices facilitating bedside assessment of automated pupillary parameters [4, 8]. Several analyses have demonstrated correlations of quantitative pupillometry readings both with ICP levels and with clinical outcomes [4, 5, 8, 9, 17]. However, all these studies used automated pupillometry as an add-on tool to invasive ICP monitoring aiming at identifying patients with ICP elevation, yet failed in establishing clear correlations with increased ICP [4, 8]. Moreover, the prognostic performance of automated pupillometry readings, and notably its clinical significance, remained unestablished, as valid stand-alone device-based measures were restricted to scenarios with imminent herniation and already pathological manual testing [4, 8]. In addition, those studies linking automated pupillometry to clinical outcomes also required further diagnostic tools to demonstrate a tentative clinical value of automated pupillometry [4, 8, 16]. Finally, all previous studies mixed patients with different neurovascular diseases or traumatic brain injury, and none of the studies accounted for co-medication, such as sedatives and vasopressors, which are known to interfere with the pupillary arc of the Central Nervous System [18]. Hence, up to now, a definite additional clinical value of automated pupillometry in the setting of neurocritical care patients remained to be verified.

In line with previous analyses, we found best discriminative thresholds of constriction velocity < 0.8 mm/s and percentage change of aperture < 10% for association with ICP elevation [9]. Yet, the prognostic value of pupillary parameters for identification of patients with ICP elevation seems to be small at all, if not absent, as positive predictive values of all dynamic pupillary parameters ranged less than 10% only. Even if CT findings with midline shift are added to this model, specificity to identify patients with definite ICP elevation ranged no higher than 65%; hence, both techniques seem not sufficient to noninvasively indicate ICP elevation. These data may explain why previous studies were unable to verify automated pupillometry readings—in the absence of further diagnostics—to reliably indicate ICP elevation. The story of a clinical management benefit flies the other way around.

We here establish a high prognostic value of pupillary parameters for identification of patients without ICP elevation, specifically in the setting of absent parallel CT imaging. A negative predictive value of 99.2% for constriction velocity adequately identifies ICH patients without ICP elevation. The clinical implications are obvious. Utilizing our constriction velocity-based threshold in clinical routine harbors a coin-flip chance of identifying patients without ICP elevation. This, however, is not at least clinically irrelevant. It means that 50% of all sedated and ventilated ICH patients who do have received prior invasive ICP measurement can now be monitored prospectively and noninvasively. The automated pupillometry device robustly identifies patients without ICP elevation, thus facilitating routine management in latter by saving unnecessary invasive ICP measurements, or repeated CT imaging, respectively.

It is well known that several clinical conditions, medications, but also physiological parameters influence pupillary reactivity [18]. Therefore, pupillary modulation may be altered in our neurocritical care patients. It has been demonstrated that the resting diameter is under sympathetic control and that sympathetic contribution to pupil size is absent during general anesthesia [19]. Although sedative and analgetic medication may also influence parasympathetic modulation [18], dysfunction of brainstem and midbrain centers mediating the parasympathetic branch of the pupillary light reflex has been shown to be particularly sensitive to compression [5]. Therefore, parameters reflecting parasympathetic pupillary modulation [7] seem most reliably associated with ICP levels; hence, future efforts should focus on establishing algorithms accounting for these findings [5].

Accurate determination of ICH patients without ICP elevation facilitates efficient and standardized management of ICH patients, as current guidelines do not provide both distinct recommendations about invasive ICP monitoring nor clear thresholds, or pharmacological interventions, for ICP treatment [2]. Thus, in the majority of ICH patients without hydrocephalus-based EVD placement, clinicians are left with frequent neurological and neuroradiological assessments to identify ICH patients at risk of clinical deterioration due to ICP elevation [1, 2]. Although specific protocols have been published suggesting algorithms for management of severe traumatic-brain-injury patients in the absence of ICP monitoring [20], our findings indicate that neither pupillometry nor neuroimaging may reliably identify ICP elevation in ICH patients. Yet, our study results may provide additional guidance in clinical management and hint toward a time- and cost-effective patient care in the future. Constriction velocity can be easily assessed at the bedside by nursing personnel and serves as a reliable, noninvasive monitoring technique in order to distinguish between patients in need of further care, i.e., imaging and eventually invasive monitoring, *versus* those who do not require such procedures. Prospective study protocols in larger cohorts are needed to validate the results of our patient population. Moreover, further research is needed to specify findings between patients with deep vs. lobar ICH. In our study cohort, ICP elevation occurred only in patients with deep ICH. We ascribe this finding to anatomical factors, as brain edema may more rapidly affect ICP in deep location ICH, and IVH as an independent risk factor for ICP elevation occurs more frequently in patients with deep location ICH [21]. Yet, our study cohort was too small to perform subgroup analyses adjusting for specific lesion size and location.

This study has certain strengths and several limitations. We here for the first time explored the prognostic performance of automated pupillometry demonstrating a clinical benefit of automated pupillometry as a stand-alone tool for routine management, while all previous studies investigated automated pupillometry as additional diagnostic maneuver only. Moreover, we focused on patients receiving sedatives and catecholamines both of which represent strong confounders of the pupillary reactivity not accounted for in previous studies. Hence, our findings that automated pupillometry might not reliably identify ICP elevation in neurocritical care patients are of clinical relevance, beyond the subgroup of patients with supratentorial ICH only. Yet, obvious limitations undermine generalizability of our results. Notably, the specific thresholds obtained by automated pupillometry (i.e., CV < 0.8, percentage change of aperture < 10%, latency > 0.3, dilation velocity < 0.2) may so far be difficult to utilize in clinical practice. Although the output of automated pupillometers comprises exact parameter values with decimal place accuracy, specific thresholds must be validated in prospective trials before they may be generalized for clinical utilization. In manual testing, we did not specify between sluggish and normal pupillary reactivity and only patients with absent light reflex were excluded from the study. Pupillometry readings also prior to EVD placement may clarify whether this method harbors the potential to identify patients in need of invasive ICP measurement. Therefore, further research is needed to implement the technique as a standard operating procedure within the initial treatment at an emergency department. For a prospective randomized study design, a delineated protocol with standardized timing of automated pupillometry, simultaneously assessed ICP along with pre-specified cranial CT scanning time points may rule out residual bias by indication and repeated measures. Prospectively assessed, time-point standardized pupillary measurements with quantitative serial assessments of pupillary function and standardized follow-up evaluation might contribute to predicting possible pupillary disturbances prior to ICP elevation. Despite the large number of pupillary assessments, the sample size of our patient group may have been too small and both groups might have been too dissimilar with respect to the number of assessments to establish new algorithms for detection of patients with increased ICP. Further, we did not stratify according to different lesion locations (i.e., lobar vs. deep), IVH and ICH volumes, respectively, all of which may vary in their susceptibility in altering pupillary function. Finally, we did not correlate automated pupillometry findings with clinical outcomes after ICH; that is why clinically relevant associations of automated pupillometry reading, other than with ICP, may have been missed [8, 16].

## Conclusion

Automated pupillometry shows associations between pupillary reactivity and ICP levels in sedated neurocritical care patients with supratentorial ICH. The clinical benefit of automated pupillometry appears rather limited for identifying ICP elevation. Yet, automated pupillometry reliably determines ICH patients without ICP elevation, thus facilitating routine management by saving invasive ICP monitoring or repeated CT controls in those patients. Prospective studies need to replicate these findings in order to verify whether automated pupillometry harbors the potential for opening up avenues for a time- and cost-effective clinical decision making in ICH patients.

## Data Availability

All data generated and analyzed during this study are included in this published article

## Abbreviations

ICP: Intracranial pressure
ICH: Intracerebral hemorrhage
CV: Constriction velocity
DV: Redilation velocity
per-change: Percentage change of apertures
Lat: Light reflex latency
CT: Computed tomography
ICU: Intensive care unit
EVD: External ventricular drain
GCS: Glasgow Coma Scale Score
NIHSS: National Institute of Health Stroke Scale score
IVH: Intraventricular hemorrhage
SD: Standard deviation
ROC: Receiver operating characteristics
AUC: Area under the curve
CI: Confidence interval
OR: Odds ratio
IQR: Interquartile range
AAPP: Automatically assessed pupillary parameters
TIA: Transient ischemic attack
mRS: Modified Rankin scale
